# The Eastern Québec Telepathology Network: a three-year experience of clinical diagnostic services

**DOI:** 10.1186/1746-1596-9-S1-S1

**Published:** 2014-12-19

**Authors:** Bernard Têtu, Émilie Perron, Said Louahlia, Guy Paré, Marie-Claude Trudel, Julien Meyer

**Affiliations:** 1 Pathology department, Centre Hospitalier Universitaire [CHU] de Québec, and Faculty of Medicine, Université Laval, Québec, Canada; 2 Pathology department, Centre Hospitalier Régional de Rimouski, Rimouski, Québec, Canada; 3 HEC Montréal, Québec, (GP, MCT & JM), Canada

## Abstract

**Background**

The Eastern Quebec Telepathology Network (called Réseau de Télépathologie de l'Est du Québec in French) was created to provide uniform diagnostic telepathology services in a huge territory with low population density. We report our first 3-year experience.

**Methods**

The network was funded equally by the Québec ministry of Health and Canada Health Infoway, a federal telehealth funding agency. The coverage includes intraoperative consultations (IOC), expert opinions, urgent analyses and supervision of macroscopic description. The deployment of the equipment and software started in 2010 and clinical activities began in January 2011. This network comprises 24 hospitals providing oncologic surgery, of which 7 have no pathology laboratory and 4 have a pathology laboratory but no pathologist. The real-time gross evaluation during IOC was performed using a macroscopy station and the sample selection was performed distantly by a technician, a pathology assistant or the surgeon under on-site pathologist supervision. Slides were scanned into whole-slide images (WSI).

**Results**

As per March 2014, 7,440 slides had been scanned for primary/urgent diagnosis; 1,329 for IOC cases and 2,308 for expert opinions. A 98% concordance rate was found for IOC compared to paraffin material and the average turnaround time was 20 minutes. Expert opinion reports were signed out within 24 hours in 68% of cases and within 72 hours in 85%. A recent multi-method evaluation study of the Network demonstrated that, thanks to telepathology: 1. interruption of IOC service was prevented in hospitals with no pathologist on site; 2. two-stage surgeries and patients transfers were prevented according to surgeons and pathologists; 3. retention and recruitment of surgeons in remote hospitals were facilitated; and 4. professional isolation among pathologists working alone was reduced. This study also demonstrated that wider adoption of telepathology would require technological improvement and that the sustainability of the network requires better coordination and the development of a supra-regional pathology organisation.

**Conclusion**

The Eastern Quebec Telepathology Network allowed the maintenance of rapid and high quality pathology services in more than 20 sites disseminated on a huge territory. A second phase is underway to expand telepathology to other regions across the province.

## Background

The Eastern Quebec Telepathology Network (called "Réseau de Télépathologie de l'Est du Québec" in French) was created in 2004 upon request from the Québec Ministry of Health to develop new telehealth initiatives in the province. It was mainly aimed at providing IOC everywhere and at all times and achieving gains in terms of the speed and quality of surgical services in a territory of 408,760 km^2 ^with 1.7 million inhabitants where the density, in certain areas, is as low as 0.4 inhabitants/km^2 ^[1]. In 2007, the Québec Ministry of Health and Canada Health Infoway, a federal telehealth funding agency, agreed to equally fund the project. Following a rigorous selection process, the deployment of the telepathology equipment and software started in 2010 and clinical activities began in January 2011.

The Network comprises 24 hospitals providing oncologic surgery, of which 21 are fully operational. Of those 24 sites, 7 have no pathology laboratory, 4 have a pathology laboratory but no pathologist and there is only one practicing pathologist in one fourth of the sites. The other 7 sites have between 2 to 15 pathologists on site. The Network is aimed at covering IOC, expert opinions, primary diagnosis/urgent analyses and macroscopy supervision. The selection of these applications was the result of a survey sent to health professionals of the concerned hospitals. Results of this survey showed that surgeons practicing in remote hospitals without a full-time on-site pathologist needed more consistent pathology coverage. These surgeons complained that without a pathologist on site, pathology coverage depended on the presence of a part-time pathologist or cases needed to be sent to a remote laboratory. They also pointed out that surgeries requiring an IOC had to be grouped on the days when the pathologist was present, thus significantly limiting the flexibility of their operating schedule. The survey also highlighted the challenge of recruiting younger pathologists who felt insecure working alone, mainly because of the difficulty to obtain a quick expert opinion on complex cases. Finally, certain community hospitals did not have enough surgical activities to justify the presence of a full-time pathologist or even of a pathology laboratory. The present article shares the results of our first three-year experience of telepathology diagnostic services.

## Methods

**Figure 1:**
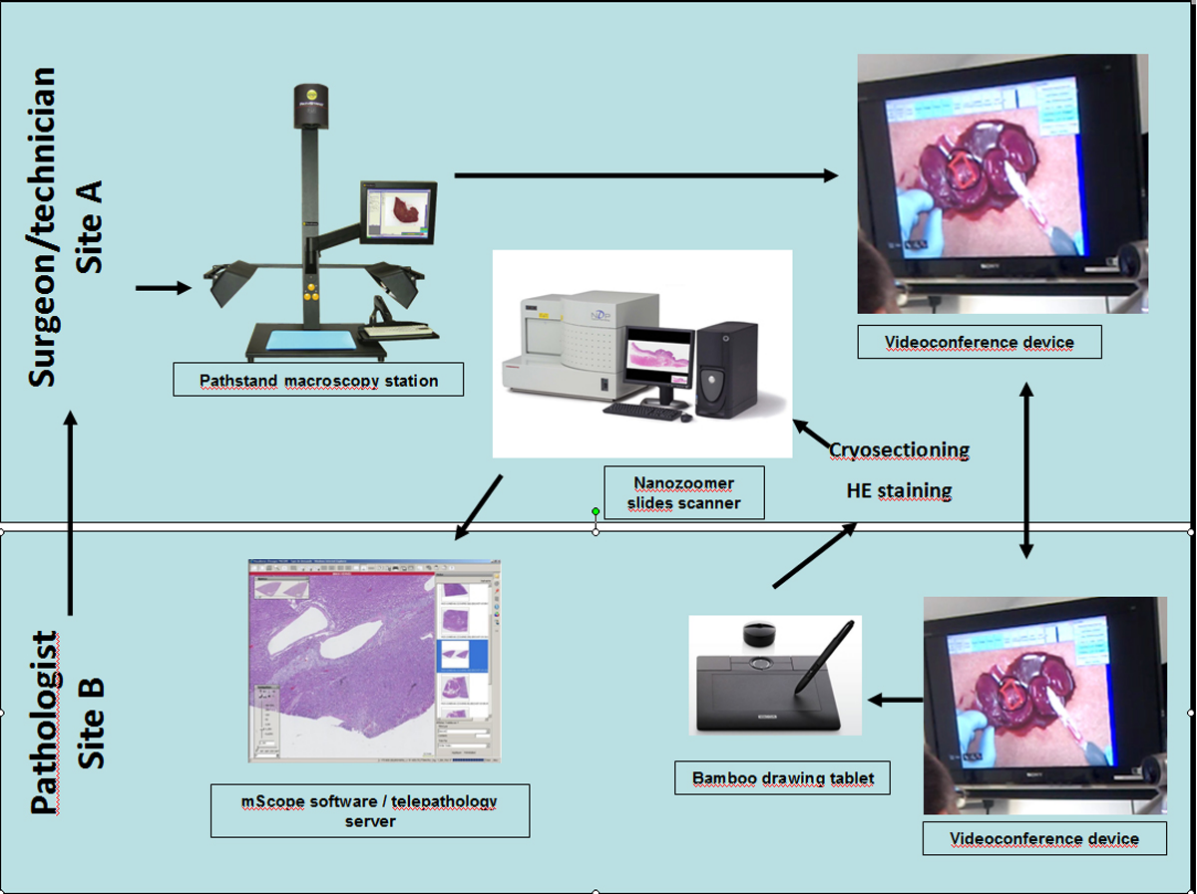
**Equipment deployed in the Network**. During an IOC, the surgeon, in site A, communicates with the pathologist in site B and shows the specimen to be analyzed via the macroscopy and videoconferencing system; the pathologist indicates to the surgeon, via the videoconferencing system, with the use of the drawing tablet, where to take the section for the histologic examination; the technician who is at the same site as the surgeon, proceeds to sectioning, staining and scanning of the slide and the pathologist examines the histology slide on his monitor and contact the surgeon via telephone to communicate his final diagnosis.

The equipment deployed in each of the 21 operational sites is shown on Figure 1 and includes a macroscopy station (PathStand 40, Diagnostic Instruments, *Sterling Height, USA*) and two videoconferencing devices (PCS-XG80DS Codec, Sony, *Minato, Tokyo, Japan*) equipped with a drawing tablet (Bamboo CTE-450K, WACOM, *Otone, Saitama, Japan*). Each site was also equipped with either a Nanozoomer 2.0 RS (16 sites) or an HT (8 sites) digital whole-slide scanner (Hamamatsu Photonics, *Shizuoka Prefecture, Japan*) and the images are saved on a local dedicated telepathology server. These pieces of equipment were obtained from Olympus Canada Inc. (*Markham*, Canada). The WSI are visualized at a 1680 × 1050 pixels resolution with the mScope v.3.6.1 (Aurora Interactive Ltd., *Montreal, Canada*) software. An additional server with an academic mScope solution was also included in the package to allow the pathologists of the Network to develop teaching activities.

For the IOC and macroscopic supervision, real-time macroscopic evaluation of surgical specimens is required. As shown on Figure 1, the macroscopy station and videoconferencing device allow the remote pathologist to interact with the surgeon, during an IOC, or with the technician/pathology assistant during a gross description to orient the selection of the area to be microscopically examined. The remote pathologist assists the specimen selection by drawing on the screen, via the drawing tablet. Once the selection of the sample is completed, the technician proceeds to cryosectioning and staining. For the IOC, primary diagnosis/urgent analyses and expert opinions, digital WSI of microscopic slides are obtained by scanning at a 20× or 40× magnification and the images are saved on the local dedicated telepathology server. Through the mScope software, the remote pathologist can read the clinical information and examine the WSI. The pathologist can also either use mScope to dictate or type a final report or use their local laboratory information system.

Since the beginning of the clinical activities in January 2011, a number of rigorous evaluations have been performed to: 1. Assess the concordance rate of the diagnosis rendered by telepathology compared to the microscope; 2. Assess the turnaround time of the diagnosis made by telepathology for IOC and expert opinion and 3. Assess the effects of the deployment of telepathology on the health care professionals, patients and on the regional organization and delivery of care. This article summarizes the findings obtained during the first three years of system use.

## Results and discussion

As per March 2014, 7,440 slides had been scanned for primary diagnosis/urgent analyses; 1,329 for IOC cases and 2,308 for expert opinions. Most IOC were from breast cancers (sentinel lymph nodes, margin close to breast cancer), lung cancer (bronchial margins, mediastinal lymph nodes) and from ovarian, pleural, peritoneal, omental lesions and from stomach and head and neck cancers (Moh's surgery). In addition, a total of 1,260 sessions of macroscopy supervision have been performed. Several smaller laboratories in community hospitals which don't have complete immunohistochemical facilities requested immunohistochemical analyses from larger laboratories. Results were occasionally returned by telepathology to obtain faster results. Although not used extensively yet, telepathology offers an interesting alternative to improve turnaround time in such situation. Teaching cases have also been shared through the mScope academic solution to allow pathologists across the Network to participate to continuing medical education and quality assurance activities.

Quality assurance is an important part of the activities of the Network. A steering committee oversees all activities of the Network, including quality assurance. Before any implementation, all potential users are being trained to use the technology. Policies have been developed regarding the indications and contra-indications of telepathology for IOC. A troubleshooting process for both the macroscopy station and the WSI system has been implemented and is being performed every morning before the beginning of IOC. Performance parameters (turnaround time, concordance studies, deferred cases) are documented. The possibility of implementing a systematic process to regularly review a number of telepathology cases is being developed as part of the Québec Quality Assurance plan. A recent quality assurance investigation showed a 98% concordance rate between the diagnosis made on the frozen material of the IOC cases compared to the final diagnosis rendered on paraffin material [2]. This concordance rate compares favorably with the situation when both the surgeon and the pathologist are at the same site [3,4]. The average turnaround time of IOC cases was 20 minutes and met the College of American Pathologists' recommendation when both the surgeon and the pathologist share the same site [5]. Expert opinion reports were signed out within 24 hours in 68% of cases and within 72 hours in 85%, which is well within the recommendations of the Association of Directors of Anatomic and Surgical Pathology [6,7]. In other words, telepathology allowed to maintain the same level of quality required in the practice of surgical pathology.

Furthermore, a recent multi-method evaluation [8] study of the Network was performed to better understand the expected and unexpected effects of telepathology on health care professionals and patients as well as on the regional organization and delivery of surgical services. Four major benefits of the introduction of telepathology have been identified. First, the interruption of IOC service was clearly prevented in hospitals with no pathologist on site. In two remote pathology laboratories, a pathologist has been on-site for more than 10 years and moved to another laboratory in two months of notice. To maintain the surgical activities requiring IOC, the only option was to obtain support from a remote pathologist by telepathology. This support was provided which allowed the continuation of the surgical activities. Second, surgeons who were interviewed mentioned that two-stage surgeries and patient transfers were prevented by telepathology. This benefit was expected for hospitals which pathology laboratory lacked a pathologist on site but was also wished in the 4 hospitals devoid of pathology laboratory and where the surgeons never had access to this service. In one of the latter, over 98 slides had been scanned for IOC, less than one year after system implementation, demonstrating the existence of such a need. Third, retention and recruitment of surgeons in remote hospitals were both facilitated. Our observations revealed at least one case of staff recruitment and one instance of staff retention in remote hospitals, thanks to the deployment of telepathology. Fourth, professional isolation and insecurity among pathologists working alone was reduced. Over 2,000 slides were submitted for expertise from such pathologists since the launch of the clinical activities in January 2011. Pathologists agreed that wider adoption of telepathology for clinical use would require improvement of current technologies, mainly in connection with response time and the ergonomics of the current software. Furthermore, the sustainability of such a network would need better coordination between the different hospitals of the Network. To be fully operational, a telepathology Network would require the creation of a regional or even a supra-regional organisation which would allow pathologists from any of the participating sites to share urgent and difficult cases. The recent evaluation of the network pointed out the gap between the overall objective of the network to offer consistent pathology coverage in a region and the legal requirement for each institution to prioritize its own in-house cases and to meet defined turnaround times. It seems clear that such technology will force different jurisdictions around the world to redefine the routing and prioritization of most urgent surgical pathology cases and adopt a more integrated and comprehensive pathology coverage at a regional or national level.

The Eastern Québec Telepathology Network is currently the most ambitious telepathology project in Canada and ranks among the most important in the world in terms of both the number of sites and geographic coverage [9]. The data collected since the implementation of the Network and summarized in this article confirm that telepathology helps to improve the accessibility and quality of surgical services in remote regions, particularly for oncological surgeries. Our experience also confirms, as reported by others [10], that the overall diagnostic review by WSI was not inferior to microscope slide review. Furthermore, data reported in the present study reveals that telepathology played a key role to support pathologists working alone and to ensure their retention in remote hospitals. Indeed, it is estimated that 10 to 20% of oncologic cases must be validated by more than one pathologist [11] and we demonstrated that telepathology is a fast and efficient method to reach this objective among pathologists practicing far from academic centers. Finally, our Network also allowed isolated pathologists to participate to online academic seminars and activities organized by academic pathologists. Current literature shows that telemedicine may help to retain physicians in remote regions by contributing to provide better working conditions [12-14]. The access to expert opinions and continuing medical education activities also ranks among those improved conditions.

However, despite the clear advantages of introducing telepathology in the daily pathology practice, there is still resistance from many pathologists and surgeons to adopt the digital technology. We identified a number of barriers to this adoption and several major legal, reimbursement, and licensure issues have already been addressed. It is clear, however, that human factors relating to the fear of using a new technology rank among the most important limitations which explains such inertia in many laboratories, even in academic institutions [1]. However, the key to the success of telepathology requires a strong communication plan and a highly coordinated effort between surgeons, pathologists, stakeholders, laboratory staff, biomedical, administrative and IT support teams working on different sites. In our network, a central coordination center financially supported by the Québec Ministry of Health has been created and each site is being visited regularly or invited to participate to follow-up videoconferences. In the past year, major steps have been completed to improve the adoption of the technology by the pathology community. A guideline on the validation process of WSI for diagnostic purposes in pathology has been recently released by the College of American Pathologists [15] and the Canadian Association of Pathologists mandated a group of Canadian experts to develop a series of guidelines to establish a telepathology service[16]. Image storage and archiving is also a major issue because of the large size of WSI. Initially, since IOC are being systematically controlled on paraffin material shortly after the surgery, it was planned to save WSI for a limited period of time only. However, a legal advice recommended applying the same retention schedule for WSI as for slides and paraffin blocks. Currently, all images are being saved and different alternatives for permanent long-term storage in our Network are under investigation.

Digital pathology has been successfully implemented in many countries around the world for education, clinical pathological conferences, and research [17]. Its adoption for diagnostic purposes is increasing, but there are still few examples of structured patient-centered networks [18,19] largely because of the many barriers that need to be overcome [20]. Canada has been a world leader in the implementation of telepathology and, recently, several companies obtained a Health Canada Class II Medical Device License for creating, managing, storing, annotating, measuring, and viewing digital whole-slide images for routine pathology [21]. Such leadership may be attributed to the initiative of a few leading individuals and to the financial support of provincial governments and Canada Health Infoway. However, it is clear that the demographic, geographic and situational characteristics of Canada, such as its immense territory, its dispersed population and the severe shortage of anatomical pathologists may explain, at least in part, the expansion of telepathology in this country.

## Conclusion

In short, our experience demonstrates that the Eastern Quebec Telepathology Network allowed the maintenance of rapid and high quality pathology services in a network of more than 20 sites dispersed across a large territory. A second phase is underway and is aimed at expanding the service to other regions in the province. It is our contention that telepathology provides otherwise unavailable services to remote communities, allows greater flexibility in pathology practice, avoids unnecessary travel and facilitates a better organisation of clinical work in a vast territory with a shortage of pathologists.

## List of abbreviations used

IOC: intraoperative consultations; WSI: Whole-slide image

## Competing interests

The authors declare that they have no competing interests.

## Authors' contributions

Bernard Têtu coordinated the project as medical director of the Network; Emilie Perron was responsible of the concordance study; Said Louahlia was responsible of coordinating the IOC concordance study: Guy Paré, Marie-Claude Trudel & Julien Meyer conducted the multi-method evaluation study.

## Notes

Publication of this supplement has been funded by 12th European Congress on Digital Pathology. This article has been published as part of *Diagnostic Pathology *Volume 9 Supplement 1, 2014: Selected articles from the 12th European Congress on Digital Pathology. The full contents of the supplement are available online at http://www.diagnosticpathology.org/supplements/9/S1
